# The Medical Problem of To-Day

**Published:** 1951-04

**Authors:** C. T. Andrews

**Affiliations:** Consulting Physician to Cornwall Geriatric Unit.


### The Bristol Medico-Chirurgical Journal

*A Journal of the Medical Sciences for the*

### West of England and South Wales

*"Scire est nescire, nisi id me*
*Scire alius sciret."*

APRIL, 1951

### GERIATRICS.*

### I: THE MEDICAL PROBLEM OF TO-DAY

BY

C. T. ANDREWS, M.D., F.R.C.P.
*Consulting Physician to Cornwall Geriatric Unit.*

The problem of the aged sick in the community to-day is urgent but complex. I propose to consider certain salient features only.

*The Population Trend.* People are living longer to-day than they did fifty years ago. In this country at the beginning of the century there were about 2½ million people over the age of sixty; to-day there are 7½ millions, and it is estimated that in 1977 there will be 10½ millions. But these figures do not represent the full measure of the problem. For the aged, as we all know, are more prone to incapacitating ailments than the rest of the community and their ailments tend to be chronic or to require prolonged convalescence. From this two results follow directly: first, an increased load is thrown on the home where someone, usually a daughter, has to be responsible for the sick parent or grandparent; secondly, the hospital service, which

* Discussion by the Bristol Medico-Chirurgical Society on Wednesday, 13th December, 1950.

quantitatively at any rate, may have been adequate for the year 1900, is totally inadequate for 1950; and unless the problem is tackled energetically it will have reached the stage of crisis long before 1977.

*The Domestic Load.* I want to emphasize the effect of these trends on the domestic structure to-day. Sheldon, in a survey of the problem in Wolverhampton, found that 7.7 per cent. of the old people seen were causing such strain on the home that the individual mainly responsible for nursing care was turned into a drudge. Here is a group indeed for whom we might say it is the clear duty of the state to take responsibility by providing accommodation for them in hospitals, homes or hostels. But you may measure this aspect of our problem to some extent when I say that were the state to provide institutional care for this group we should have to increase our present accommodation fourfold.

*The Hospital Problem.* The hospital side of the problem is familiar to everyone. Nevertheless it may be worthwhile to look at it statistically as we find it in an isolated geographical area such as West Cornwall. And first we must not forget that every hospital and every speciality (excepting those dealing with obstetrics and pediatrics) is directly concerned, though some of course more than others. On a single day in Cornwall it was found by taking a census, that the aged population in hospitals of all sorts represented 2.5 per cent. of the total, only 0.9 per cent. being in hospitals of the geriatric service. On the same day 40 per cent. of all occupied beds were occupied by patients over sixty.

Now all this adds up to the fact that the hospitals are dealing on an increasing scale with the ailments of old age. Much of this is as it should be, but much is due to inadequacy of the service being provided in the special hospitals for this age-group. So let us look for a moment at these hospitals for the aged sick. For it is certain that here is the crux of the problem so far as the present day is concerned.

The provision of institutional care in this country dates mainly from the early part of the nineteenth century. These grim grey buildings were originally intended to cope with destitution. Gradually parts of them were converted to hospital wards and in these nursing and a mnimium of medical care was provided. The buildings so adapted were often entirely unsuitable for hospital purposes. All types of patients might be found in one building and even in one ward. There were no X-ray or laboratory facilities, no consultant staff. The shortage of nursing staff was often acute though the quality of nursing care was good. One result of this was that the patients were put to bed where they were out of the way and safe. Up and about they tripped over things and fell downstairs. So they were kept in bed and gradually their limbs and abdominal muscles atrophied and then contracted. Their joints became ankylosed, they developed kyphosis. Mental apathy followed by senile dementia with incontinence completes the picture of thousands of old people in these hospitals to-day.

To remedy this state of affairs in a clinical area such as West Cornwall action is required along three lines:

(*a*) Careful examination and classification of patients.

(*b*) The establishment of an active rehabilitation centre.

(*c*) Segregation of various groups such as the psychiatric patients and the long-stay-irremediable.

The most serious difficulty arises with the frail ambulant patient, who is scarcely fit for the rigorous life in an institution, but for whom the local authority may not be able to find hostel accommodation.

Economic and other considerations have led both in this country and America to serious attempts to provide a domiciliary service which will parallel in efficiency that provided by the hospital. The ingredients of such a service are to hand in this country in the form of the specialist domiciliary service, the general practitioner, the nursing service, home helps, free laundry, mobile meals and physiotherapy. A number of different authorities, however, are concerned, and some co-ordinating body is needed to bring together all the agencies working on behalf of old people.

The necessity for educating the medical student in the clinical and social aspects of this problem is apparent when the population trend is considered. The aged must be maintained in health and employment as long as possible. Indeed it may be said that if the achievement of medicine in the last fifty years has been that of adding years to the life of man the problem of the next fifty is that of adding life to his years.

Yet it would be a mistake to regard this as altogether a problem in social medicine. Geriatrics, like pediatrics needs a sound basis in research and clinical observation. From that point of view we can hardly say we have made a beginning. And one of the greatest dangers of the hour is that the necessity for a sound clinical background may be lost sight of in the welter of social problems which are constantly arising.

REFERENCES

Sheldon, J. H.—*The Social Medicine of Old Age* (Nuffield Foundation, 1948).

Bluestone, E. M.—" Long Term Illness and the Hospital of the future." *Bulletin Am. Coll. of Surgeons*, September, 1949.

*Ibid.*—" The Place of the Long-Term Patient in the Modern Hospital." *Bulletin Am. Coll. of Surgeons*, June, 1946.

*Ibid.*—*Survey*, 112 *East* 19 *St.*, *New York*, April, 1948.

